# Impact of smart technology use on sleep quality in individuals with autism spectrum disorder: a mixed-methods investigation

**DOI:** 10.3389/fpsyt.2024.1411993

**Published:** 2024-05-24

**Authors:** Mohamed Abouzed, Basem Salama, Amgad Gabr, Khaled A. Elag, Mahmoud Soliman, Nisrin Elsaadouni, Nasr Abou Elzahab

**Affiliations:** ^1^ Psychiatry Department, Faculty of Medicine, Al-Azhar University, Cairo, Egypt; ^2^ Community Medicine Department, Faculty of Medicine, Northern Border University, Arar, Saudi Arabia; ^3^ Psychiatry Department, Faculty of Medicine, Ain Shams University, Cairo, Egypt; ^4^ Neuropsychiatry Department, Faculty of Medicine, Ain Shams University, Cairo, Egypt; ^5^ Psychiatry Department, Faculty of Medicine, Mansoura University, Mansoura, Egypt; ^6^ New Damietta, Faculty of Medicine, Al-Azhar University, New Damietta, Egypt

**Keywords:** autism spectrum disorder, sleep quality, smart technology, screen time, blue light exposure, mixed methods

## Abstract

**Background:**

Sleep disturbances are common among individuals with autism spectrum disorder (ASD) and can have a negative impact on their daily functioning and core symptoms. As the use of smart technologies continues to rise, it is crucial to understand how these devices affect the sleep quality of individuals with ASD.

**Aim:**

The objective of this study was to examine the relationship between the use of smart technology and sleep quality in individuals with ASD.

**Methods:**

A mixed-methods approach was employed, combining both quantitative and qualitative data collection techniques. A sample of 83 individuals with ASD, aged between 8 and 25 years, assessed their sleep quality using the Pittsburgh Sleep Quality Index. Additionally, information regarding patterns of smart technology use and relevant covariates was collected. Correlation and regression analyses were conducted to analyze the data. Furthermore, semi-structured interviews were conducted with a subset of participants and their caregivers.

**Results:**

Significant positive correlations were found between poorer sleep quality scores and total screen time (r = 0.42, p < 0.001), pre-bedtime technology use (r = 0.51, p < 0.001), gaming (r = 0.32, p = 0.003), and social media use (r = 0.29, p = 0.008). Pre-bedtime technology use was a significant predictor of poorer sleep quality (β = 0.32, p = 0.006), even after controlling for age, gender, and ASD severity.

**Conclusion:**

The findings of this study emphasize the significant associations between the use of smart technology, particularly before bedtime, and poorer sleep quality in individuals with ASD. These results underscore the importance of developing evidence-based interventions and guidelines to promote healthy sleep habits and mitigate the negative effects of technology exposure in this population.

## Introduction

Autism spectrum disorder (ASD) is a neurodevelopmental condition characterized by persistent challenges in social communication and interaction, as well as restricted and repetitive patterns of behavior, interests, or activities (1). One of the common co-occurring issues faced by individuals with ASD is sleep disturbances, which can manifest as difficulties falling asleep, frequent nighttime awakenings, and poor sleep quality (2, 3). Sleep problems in ASD have been associated with a range of negative outcomes, including exacerbation of core autism symptoms, impaired daytime functioning, and decreased quality of life for both individuals and their families (4, 5).

In recent years, the proliferation of smart technologies, such as smartphones, tablets, and computers, has become increasingly prevalent in daily life, including among individuals with ASD. While these devices can offer educational and entertainment value, there are concerns about the potential impact of their use, particularly the blue light emission and stimulating content, on sleep quality (6–8). Preliminary research suggests that excessive screen time and exposure to blue light before bedtime may disrupt the natural sleep-wake cycle and melatonin production, leading to sleep onset difficulties and fragmented sleep (9–11).

The impact of utilizing smart technology on sleep quality can be comprehended by examining it through the lens of chronobiological and cognitive-behavioral frameworks. According to chronobiological theories, exposure to blue light emitted from electronic device screens can hinder the secretion of melatonin and disturb the circadian rhythms that regulate sleep-wake cycles (9). Melatonin, a hormone produced by the pineal gland, plays a vital role in initiating sleep and ensuring its quality. Exposure to blue light, especially during the evening hours, can delay the increase in melatonin levels, making it more challenging to fall asleep (11–17).

Moreover, the cognitive-behavioral model of insomnia (18) suggests that engaging in stimulating or arousing activities, such as gaming or consuming social media content before bedtime, can heighten cognitive and physiological arousal, thus making the transition into sleep more difficult. This model proposes that individuals with insomnia develop maladaptive habits and thought patterns related to sleep, perpetuating the sleep disturbance (19).

For individuals with autism spectrum disorder (ASD), the impact of smart technology on sleep quality may be particularly significant. Many individuals with ASD experience challenges in sensory processing (20), which could make them more sensitive to the stimulating effects of blue light and electronic content. Additionally, the core symptoms of ASD, such as difficulties with transitions and insistence on routines, may be exacerbated when technology disrupts bedtime routines (2). Indeed, preliminary research has indicated associations between increased screen time and poorer sleep quality in children and adolescents with ASD (12, 13).

Despite the high prevalence of sleep disturbances in individuals with ASD and their profound impact, there is limited research exploring the specific role of smart technology use on sleep quality in this population. While a few studies have identified associations between increased screen time and poorer sleep outcomes in children and adolescents with ASD (12, 13), a more comprehensive understanding of the underlying mechanisms and potential mediating factors is necessary. The present study aims to address this research gap by investigating the relationship between patterns of smart technology use and sleep quality in individuals with ASD across a wide age range (8–25 years). By examining variables such as total screen time, technology use before bedtime, types of content consumed (e.g., gaming, social media), and exposure to blue light, this study seeks to identify potential mediators that may influence the impact of technology on sleep quality. Understanding these mediating factors could inform the development of targeted interventions and guidelines to promote healthy sleep habits and mitigate the negative effects of technology exposure in the ASD population.

## Methodology

### Research design

This investigation utilized a mixed-methods strategy, integrating both quantitative and qualitative data collection methods, to explore the effects of utilizing smart technology on the quality of sleep among individuals diagnosed with autism spectrum disorder (ASD).

### Subjects

A total of 83 individuals with ASD, ranging in age from 8 to 25 years, were recruited from 2 autism support organizations in the Cairo and Mansoura cities in Egypt.

### Participant recruitment

Participants were recruited from two autism support organizations: the Advance Society for Persons with Autism in Cairo, and the Autism Support Center in Mansoura. Both are non-profit organizations providing services and resources for individuals with ASD and their families.

Initially, administrators at these centers were approached to obtain approval for participant recruitment. Once approved, they provided a list of all registered individuals aged 8–25 who had a confirmed diagnosis of ASD according to the DSM-5 criteria.

The research team then sent information sheets explaining the study aims, procedures, and criteria to the primary caregivers of these potential participants. Interested caregivers were asked to return a reply form indicating their willingness to be contacted further about the study. Out of 175 information sheets sent, 102 positive replies were received. The research team then screened these 102 individuals based on the inclusion/exclusion criteria via telephone interviews with their caregivers.

Sixteen individuals were excluded after this screening: 8 due to having severe intellectual disability (IQ<70), 5 for having significant sensory impairments, and 3 for other coexisting conditions like sleep disorders.

Of the remaining 86 eligible individuals, 83 went on to enroll and provide complete data for the study. Three eligible individuals declined to participate due to a lack of time/interest.

Therefore, the final study sample consisted of 83 participants aged 8–25 years with ASD who were regular users of smart technology, did not have any significant comorbidities that could interfere with the study assessments, and provided complete data across all study measures. This recruitment process adhered to the STROBE (Strengthening the Reporting of Observational Studies in Epidemiology) guidelines for observational studies.

Smart Technology Use Questionnaire.

To comprehensively assess participants’ patterns of smart technology usage, a tailored questionnaire was developed by the research team. The questionnaire items covered the types of devices used (e.g., smartphones, tablets, laptops), total screen time per day, duration of use before bedtime, types of content consumed (gaming, social media, educational apps), and timing of technology use in relation to sleep.

This thorough development and pilot testing process helped ensure the questionnaire’s content validity, clarity, and feasibility of administration for assessing smart technology usage patterns in individuals with ASD. Quantitative Component

1. The assessment of sleep quality and disturbances was conducted using the Pittsburgh Sleep Quality Index (PSQI) (21). This widely used 19-item self-report questionnaire evaluates seven components of sleep, including subjective sleep quality, sleep latency, sleep duration, habitual sleep efficiency, sleep disturbances, use of sleep medication, and daytime dysfunction.

Each component is assigned a score ranging from 0 to 3, with a higher score indicating greater dysfunction. The scores for the seven components are then summed to generate a global PSQI score, which can range from 0 to 21. Higher scores on the global PSQI indicate poorer sleep quality. A global PSQI score above 5 is considered indicative of significant sleep disturbance (16).

In the current study, the PSQI demonstrated good internal consistency, as indicated by a Cronbach’s alpha of 0.82 for the global score. This finding aligns with previous research that validated the Arabic version of the PSQI in the general Egyptian population, which reported a Cronbach’s alpha of 0.857 (14). Therefore, based on its reliability and validation, the PSQI was considered a dependable instrument for assessing sleep quality in the present study involving Egyptian individuals with ASD (15).

2. To examine smart technology use, a tailored questionnaire was created to analyze participants’ smart technology usage patterns, encompassing the types of devices utilized (e.g., smartphones, tablets, laptops), duration of use (total screen time and pre-bedtime usage), timing of use (e.g., before bedtime, overnight), and content consumption (e.g., gaming, social media, educational applications). The initial draft of the questionnaire was reviewed by a panel of three experts: one psychiatrist specializing in ASD, one clinical psychologist, and one sleep medicine specialist. Their feedback was incorporated to refine the questionnaire’s content and phrasing to ensure clarity and relevance for the ASD population.

Subsequently, the revised questionnaire underwent pilot testing with a sample of 10 individuals with ASD (age range 10–22 years) and their caregivers. This pilot allowed the research team to evaluate the comprehensibility of the items and identify any potential issues or areas of confusion.

Based on the pilot testing, some items were rephrased for simplicity, and examples were added to certain questions to improve understanding. Feedback from the pilot participants and caregivers was also used to estimate the average time required for questionnaire completion.

The final version of the smart technology use questionnaire consisted of 25 items and took approximately 15–20 minutes to complete. To establish its psychometric properties in the study sample, the internal consistency reliability (Cronbach’s alpha) of the total scale and relevant subscales were calculated and found to be satisfactory (total scale α = 0.82).

3. Various standardized questionnaires were employed to gather data on potential covariates and mediating factors, such as sensory sensitivities [Sensory Experiences Questionnaire, (22)], symptoms of anxiety and depression (Beck Anxiety Inventory), (23); Beck Depression Inventory, (24).

Participants and their caregivers completed the questionnaires as outlined above, either through online platforms or in person, based on their preferences. Caregiver reports were collected for participants aged 8–17 years regarding sleep quality and technology use, while the participants themselves completed self-report measures on sensory sensitivities, anxiety, and depression. For participants aged 18 years and older, self-report measures were utilized for all assessments. Support was provided when needed to ensure the questionnaires were understood and completed accurately.

Statistical analysis involved calculating descriptive statistics to summarize the characteristics of the sample and the trends in smart technology use and sleep quality. Pearson’s correlation analyses were carried out to explore the connections between smart technology use variables (such as duration, timing, and content) and sleep quality scores on the PSQI. Multiple regression analyses were conducted to investigate the influence of smart technology use on sleep quality while considering potential confounding variables (like age, gender, sensory sensitivities, and comorbidities). Additionally, mediation analyses using the PROCESS macro for SPSS were performed to investigate potential mediating factors affecting the relationship between smart technology use and sleep quality (25).

The qualitative component of the study involved conducting semi-structured interviews with a subset of 20 participants. This subset consisted of 10 individuals aged 8–17 years and their caregivers, as well as 10 individuals aged 18–25 years. The purpose of these interviews was to delve deeper into their experiences, challenges, and perspectives regarding the impact of smart technology use on sleep.

During the interviews, various topics were explored, including sleep routines, patterns of technology usage, perceived effects of technology on sleep, and potential strategies or interventions that have been tried or could be beneficial. To ensure accuracy, the interviews were audio-recorded and transcribed verbatim.

Thematic analysis, as proposed by Braun and Clarke in 2006, was employed to identify recurring themes and patterns within the interview data. Two independent coders were responsible for analyzing the transcripts, and any discrepancies in coding were resolved through discussion to ensure reliability.

The qualitative data obtained from these interviews served to complement and enrich the quantitative findings of the study. By providing a more comprehensive understanding of the research question, the qualitative component added depth and context to the overall findings.

### Ethical considerations

The research received ethical clearance from the Review Board of Al-Azhar University and adhered to approved procedures for obtaining informed consent, ensuring data confidentiality, and safeguarding the well-being of participants. Comprehensive information about the study was shared with participants and their caregivers, and written consent was acquired before data gathering. In cases where participants were between 8- 17 years old, assent was sought from the participant along with consent from the caregiver. The data was anonymized and securely stored, with limited access granted only to authorized research staff.

## Result

### Sample characteristics

The 83 participants in the research sample, whose ages ranged from 8 to 25, had been diagnosed with autism spectrum disorder (ASD). The majority (48%) of the population was in the 13–17 age range. In line with the higher prevalence of ASD in men, the percentage of males was higher (75%) than that of females (25%). in relation to the severity of autism. Moderate severity (54%), mild (34%), and severe (12%) (see **Table 1**).

**Table 1:** Sample Characteristics.

Characteristic		n	%
Age (years	8-12	25	30%
	13-17	40	48%
	18-25	18	22%
Gender	Male	62	75%
	Female	21	25%
ASD Severity	Mild	28	34%
	Moderate	45	54%
	Severe	10	12%

### Patterns of smart technology use

Participants, on average, revealed that they spent 5.2 hours daily utilizing smart technologies, with 1.8 hours of usage taking place before going to bed. The most extensive use was observed in gaming (2.1 hours per day), followed by social media (1.5 hours per day) and educational apps (0.9 hours per day) (See **Table 2**).

**Table 2:** Patterns of Smart Technology Use.

Technology Use Variable	Mean	SD
Total screen time (hours/day)	5.2	(2.1)
Pre-bedtime use (hours)	1.8	(1.2)
Gaming (hours/day)	2.1	(1.6)
Social media (hours/day)	1.5	(1.3)
Educational apps (hours/day)	0.9	(0.7)

Correlation between Smart Technology Use and Sleep Quality

Positive correlations suggest that increased technology utilization is linked to decreased sleep quality (elevated PSQI scores). The most robust relationship was identified between technology use before bedtime and sleep quality (r = 0.51), indicating that the timing of usage could be crucial. Additionally, moderate positive connections were noted for overall screen time (r = 0.42), gaming (r = 0.32), and social media engagement (r = 0.29). Nevertheless, the link between educational application usage and sleep quality did not reach statistical significance (see **Table 3**).

**Table 3:** Correlation between Smart Technology Use and Sleep Quality.

Technology Use Variable	r	P value
Total screen time	0.42	p < 0.001
Pre-bedtime use	0.51	p < 0.001
Gaming	0.32	0.003
Social media	0.29	0.008
Educational apps	0.11	0.321

### Multiple regression analysis predicting sleep quality

The study incorporated age, gender, ASD severity, total screen time, and pre-bedtime technology use as independent variables. Despite age, gender, and total screen time not showing significance as predictors, ASD severity (β = 0.22, p = 0.033) and pre-bedtime technology use (β = 0.32, p = 0.006) were identified as significant predictors of decreased sleep quality. These findings indicate that individuals with more severe ASD symptoms and increased pre-bedtime technology use may face a higher likelihood of experiencing sleep disturbances see **Table 4**.

**Table 4:** Multiple Regression Analysis Predicting Sleep Quality.

Predictor Variable	B	SE B	β	p
Age	-0.02	-0.05	0.04	0.623
Gender	0.18	0.29	0.06	0.535
ASD Severity	0.41	0.19	0.22	0.033
Total Screen Time	0.09	0.07	0.16	0.198
Pre-bedtime Use	0.34	0.12	0.32	0.006

### Themes from qualitative interviews

The first theme, “Effects on Sleep Perception,” discusses the different ways in which the use of smart technology was perceived to have a negative impact on sleep, including difficulties in falling asleep, waking up during the night, and feeling tired during the day. The second theme, “Obstacles and Worries,” addresses the difficulties encountered in controlling technology usage, the potential for excessive stimulation from bright screens and noises, and the disturbance of established sleep patterns. The third theme, “Possible Approaches and Measures,” presents some of the strategies or measures proposed by participants and caregivers, such as setting limits on screen time, utilizing blue light filters, establishing consistent bedtime routines, and increasing parental supervision of technology usage (See **Table 5**).

**Table 5:** Themes from Qualitative Interviews.

Theme	Subthemes	Sample Quotes
Effects on Sleep Perception	Sleep onset difficulties - Nighttime awakenings - Daytime fatigue	“If my son uses his iPad late at night, he usually doesn’t go to sleep until far after midnight.” “My daughter gets up several times a night, generally after viewing videos or playing games before going to bed.” “He just gets so exhausted and grumpy during the day after having a restless night.”
Obstacles and Worries	Difficulty regulating technology use - Sensory stimulation - Sleep routine disruption	“Getting him to put down the iPad and go to bed is an ongoing struggle.” “The bright screens and sounds from games overstimulate his senses, making it impossible to wind down.” “When she uses technology late at night, it throws off her entire sleep schedule.”
Possible Approaches and Measures	Screen time limits - Blue light filters - Bedtime routines - Parental monitoring	“It’s helped a little to establish a strict ‘no screens’ rule an hour before bed.” “Using night mode or blue light filter apps might reduce the impact on his sleep.” “Having a consistent bedtime routine with relaxing activities instead of technology seems to work better.”

## Discussion

The current study aimed to explore the association between the use of smart technology and sleep quality in individuals diagnosed with autism spectrum disorder (ASD). Both quantitative and qualitative methods were employed to investigate this relationship. The findings of this study contribute to the existing body of literature on the potential impact of technology exposure on sleep, specifically within the context of ASD. This population is known to be particularly susceptible to sleep disturbances.

The results of the study revealed positive correlations between various aspects of smart technology use, such as total screen time, pre-bedtime use, gaming, and social media, and lower scores on the Pittsburgh Sleep Quality Index (PSQI). These findings align with the predictions of chronobiological theories, which suggest that exposure to blue-enriched light emitted by electronic devices, especially during the evening, can suppress the secretion of melatonin and disrupt the sleep-wake cycle. Previous research has shown that this can lead to delays in circadian phase shifts, ultimately affecting sleep quality.

Furthermore, the cognitive-behavioral model of insomnia may provide insights into the underlying mechanisms linking technology use and sleep disturbances in individuals with ASD. Activities like gaming and engaging with social media often involve stimulating content and interactive features, which can lead to increased cognitive and physiological arousal. This heightened state of arousal may make it more challenging for individuals to transition to a relaxed state conducive to sleep. The qualitative data collected in this study support this notion, as participants and caregivers reported difficulties in winding down and adhering to bedtime routines due to the overstimulating effects of technology use. Furthermore, the sensory processing challenges often experienced by individuals with ASD (5) may exacerbate the impact of technology exposure on sleep. The bright lights, sounds, and visual stimuli associated with smart device use could potentially trigger sensory overload, further disrupting the ability to self-regulate and transition to a relaxed state before bedtime.

The results of the current investigation align with previous studies conducted on neurotypical individuals, which have demonstrated the detrimental effects of evening technology use and exposure to blue light on sleep quality (7–9, 11). However, this study goes beyond previous research by examining these findings in the context of individuals with autism spectrum disorder (ASD), who may experience compounded effects due to differences in sensory processing, cognitive and behavioral rigidity, and potential difficulties with self-regulation.

The observed connections between the use of smart technology and sleep quality in individuals with ASD are consistent with the limited existing research in this field (12, 13). Nevertheless, the present study offers a more comprehensive exploration of this relationship by investigating various aspects of technology use, such as timing and content, and utilizing a mixed-methods approach that incorporates both quantitative and qualitative perspectives.

The implications of these findings are significant, as sleep disturbances in individuals with ASD have been associated with a range of negative outcomes, including the exacerbation of core symptoms, difficulties in cognition and behavior, mood dysregulation, and overall decreased well-being (4, 5). It is crucial to address the potential impact of smart technology use on sleep quality in this population in order to promote better sleep hygiene and mitigate the cascading effects of sleep disturbances on various domains of functioning.

### Potential interventions and future directions

According to Green et al. (11), it is suggested that individuals with ASD may benefit from implementing strategies such as setting technology curfews or limiting the use of light-emitting devices before bedtime, as there is a strong association between pre-bedtime technology use and poor sleep quality. Additionally, the use of blue light filters or night mode settings on devices could potentially help reduce the disruption of circadian rhythms and melatonin production.

The qualitative data also emphasizes the importance of establishing consistent bedtime routines that involve relaxing activities not involving technology. This aligns with existing recommendations for individuals with ASD, which highlight the significance of structured routines and environmental modifications in promoting healthy sleep habits (4).

In addition to behavioral interventions, there is potential for utilizing technology itself to develop solutions or interventions that can mitigate the negative impact on sleep. For example, exploring parental control apps or software that can monitor and restrict device usage during specific times could be beneficial. Similarly, wearable devices or smartphone applications that track sleep patterns and provide personalized recommendations or interventions could serve as valuable tools for this population.

Future research should prioritize addressing the limitations of the current study, which include the use of a cross-sectional design and reliance on self-reported and caregiver-reported measures. To establish causal relationships and gain a better understanding of the underlying mechanisms, it would be beneficial to conduct longitudinal or experimental studies that incorporate objective measures of technology use (e.g., smartphone tracking) and sleep (e.g., actigraphy).

Furthermore, expanding the scope of variables considered in future research, such as comorbidities (e.g., anxiety, depression), medication use, and environmental factors, would provide a more comprehensive understanding of the potential factors that mediate or moderate the relationship between technology use and sleep quality in individuals with Autism Spectrum Disorder (ASD).

In addition, it is important to conduct research specifically focused on evaluating the effectiveness of interventions or technologies aimed at promoting healthy sleep habits in individuals with ASD. Studies of this nature could assess the efficacy of strategies like blue light filters, parental control apps, or cognitive-behavioral interventions tailored to the unique needs and challenges of individuals with ASD.

### Limitations

While the current investigation offers valuable insights, it is important to acknowledge several limitations. Firstly, due to the relatively small number of participants, and secondary,due to its cross-sectional nature, the study is unable to establish causal relationships between the use of smart technology and sleep quality. Moreover, the reliance on self-reported and caregiver-reported measures of technology use and sleep quality introduces the possibility of recall bias or social desirability bias.

Additionally, the study fails to consider potential confounding factors such as comorbid conditions (e.g., anxiety, depression), medication usage, or environmental factors that may impact sleep quality. To overcome these limitations, future research should strive to address them by incorporating objective measures, employing longitudinal study designs, and conducting more comprehensive assessments of potential confounding variables.

## Conclusion

The results of this study draw attention to the significant connections between the use of smart technology, especially before bedtime, and a decline in sleep quality among individuals with autism spectrum disorder (ASD). The combination of both quantitative and qualitative data offers a thorough comprehension of the perceived consequences, difficulties, and potential strategies associated with technology use and sleep in this particular group.

These findings emphasize the necessity of establishing evidence-based interventions and guidelines to encourage healthy sleep habits and alleviate the adverse effects of smart technology use in individuals with ASD. By addressing this matter, we can promote enhanced sleep quality, which may have a ripple effect on overall well-being, behavior, and functioning within this vulnerable population.

In the future, research should concentrate on elucidating the underlying mechanisms by which technology exposure disrupts sleep, evaluating the effectiveness of specific interventions, and investigating the long-term consequences of sleep disturbances on various aspects of functioning in individuals with ASD. Ultimately, a multidisciplinary approach that involves collaboration among researchers, clinicians, and technology developers is essential to developing comprehensive solutions that tackle the intricate interplay between technology use, sleep, and neurodevelopmental disorders such as ASD.

## Data availability statement

The raw data supporting the conclusions of this article will be made available by the authors, without undue reservation.

## Ethics statement

The studies involving humans were approved by Al-Azhar University Review Board. The studies were conducted in accordance with the local legislation and institutional requirements. Written informed consent for participation in this study was provided by the participants’ legal guardians/next of kin.

## Author contributions

MA: Conceptualization, Writing – original draft. BS: Methodology, Validation, Writing – review & editing. AG: Data curation, Investigation, Writing – original draft. KE: Investigation, Supervision, Writing – review & editing. MS: Conceptualization, Investigation, Writing – original draft. NE: Investigation, Validation, Writing – review & editing. NA: Data curation, Investigation, Methodology, Writing – review & editing.
