# Supplementary material for: Impact of smart technology use on sleep quality in individuals with autism spectrum disorder: a mixed-methods investigation
Source: Front Psychiatry. 2024 May 24;15:1411993. doi: 10.3389/fpsyt.2024.1411993 (PMC11157126; doi:10.3389/fpsyt.2024.1411993)
Supplement: Supplementary file 1 [file Table_1.docx]

Smart Technology Use Questionnaire

Instructions: Please answer the following questions about your (or your child's) use of smart technologies such as smartphones, tablets, laptops, and computers.

1. On average, how many hours per day do you (or your child) spend using smart technologies (e.g., smartphones, tablets, laptops, computers)?

_____ hours per day

2. On average, how many hours do you (or your child) spend using smart technologies in the hour before bedtime?

_____ hours before bedtime

3. Which of the following smart devices do you (or your child) use regularly? (Select all that apply)

☐ Smartphone

☐ Tablet

☐ Laptop

☐ Desktop computer

☐ Other (please specify): __________________

4. On a typical day, how much time do you (or your child) spend on the following activities using smart technologies?

a. Gaming: _____ hours per day

b. Social media (e.g., Facebook, Instagram, TikTok): _____ hours per day

c. Educational apps or websites: _____ hours per day

d. Streaming videos/movies: _____ hours per day

e. Browsing the internet: _____ hours per day

f. Other (please specify): __________________: _____ hours per day

5. Do you (or your child) use any blue light filters or night mode settings on your smart devices in the evening?

☐ Yes

☐ No

6. How often do you (or your child) experience difficulties falling asleep or staying asleep after using smart technologies in the evening? (Select one)

☐ Never

☐ Rarely

☐ Sometimes

☐ Often

☐ Always

7. Have you (or your child's caregiver) tried any strategies to limit or manage smart technology use before bedtime? If yes, please describe:

_____________________________________________________________..
